# Elevated Plasma Soluble ST2 Levels are Associated With Neuronal Injury and Neurocognitive Impairment in Children With Cerebral Malaria

**DOI:** 10.20411/pai.v7i1.499

**Published:** 2022-06-23

**Authors:** Elizabeth M. Fernander, Pontian Adogamhe, Dibyadyuti Datta, Caitlin Bond, Yi Zhao, Paul Bangirana, Andrea L. Conroy, Robert O. Opoka, Chandy C. John

**Affiliations:** 1 Ryan White Center for Pediatric Infectious Disease and Global Health, Department of Pediatrics, Indiana University School of Medicine, Indianapolis, IN, USA; 2 Department of Biostatistics and Health Data Science, Indiana University School of Medicine, Indianapolis, IN; 3 Department of Psychiatry, Makerere University, Kampala, Uganda; 4 Department of Paediatrics and Child Health, Makerere University, Kampala, Uganda

**Keywords:** Cerebral Malaria, Severe Malarial Anemia, ST2, IL-33, Cognitive Impairment

## Abstract

**Background:**

Murine experimental cerebral malaria studies suggest both protective and deleterious central nervous system effects from alterations in the interleukin-33 (IL-33)/ST2 pathway.

**Methods:**

We assessed whether soluble ST2 (sST2) was associated with neuronal injury or cognitive impairment in a cohort of Ugandan children with cerebral malaria (CM, n=224) or severe malarial anemia (SMA, n=193).

**Results:**

Plasma concentrations of sST2 were higher in children with CM than in children with SMA or in asymptomatic community children. Cerebrospinal fluid (CSF) sST2 levels were elevated in children with CM compared with North American children. Elevated plasma and CSF ST2 levels in children with CM correlated with increased endothelial activation and increased plasma and CSF levels of tau, a marker of neuronal injury. In children with CM who were ≥5 years of age at the time of their malaria episode, but not in children <5 years of age, elevated risk factor-adjusted plasma levels of sST2 were associated with worse scores for overall cognitive ability and attention over a 2-year follow-up.

**Conclusions:**

The study findings suggest that sST2 may contribute to neuronal injury and long-term neurocognitive impairment in older children with CM.

## INTRODUCTION

Cerebral malaria (CM) and severe malarial anemia (SMA) are among the most devastating manifestations of *Plasmodium falciparum* infection. Globally, approximately two-thirds of malaria deaths are estimated to occur in children <5 years old, resulting in almost 600,000 deaths in Africa in 2020 [1]. In addition, CM and SMA have been associated with long-term neurocognitive impairment in children who survive CM [2–5] or SMA [6]. However, the mechanisms underlying disease severity and neurocognitive impairment in CM and SMA are not fully understood.

Microvascular obstruction, endothelial activation, and excessive systemic inflammation that occur, in part, from parasite burden and sequestration of infected erythrocytes are thought to underlie the pathogenesis of both CM and SMA [7–13]. The excessive production of pro-inflammatory cytokines is an important component of systemic inflammation during severe disease, particularly CM [10]. Interleukin-33 (IL-33), an alarmin molecule released early in response to tissue or endothelial barrier damage in several inflammatory diseases [14], is a member of the IL-1 cytokine family and binds to its receptor, ST2 [15]. ST2 has 2 splice variants: a membrane-bound form, which initiates IL-33 intracellular signaling; and the soluble form, soluble ST2 (sST2), which binds IL-33 in circulation, inhibiting the intracellular signaling of IL-33 [16]. IL-33 and ST2 are widely expressed throughout the human body, and low plasma or serum levels of IL-33 or high levels of sST2 are seen in numerous diseases, including cardiovascular diseases, particularly heart failure [17, 18] and stroke [19, 20], and infectious diseases, including sepsis [21, 22] and recently COVID-19 [23, 24]. However, elevated IL-33 has also been associated in human studies with increased endothelial activation and neuroinflammation [25, 26], suggesting that it may have a pleiotropic effect.

Studies of experimental cerebral malaria (ECM) in mice using *Plasmodium berghei* ANKA haveidentified IL-33 and ST2 as key molecules involved in ECM pathogenesis. Studies support a role for endogenous IL-33 in increasing inflammation and contributing to development of ECM [27] and associated cognitive impairments [28]. However, exogenous IL-33 has been shown to reduce inflammation and mediate protection from ECM [29, 30]. Taken together, the murine ECM studies and human studies of the role of the IL-33/ST2 pathway in infectious processes highlight the complex interplay of this pathway in the host response to infection.

In human studies, sST2, which acts as a decoy receptor to bind IL-33 and reduce the intracellular effects of IL-33, is often measured in place of IL-33, because it is present in higher levels in plasma or serum and is a better predictor of disease severity and mortality than IL-33 [16, 31]. However, plasma and central nervous system (CNS) levels of sST2 have not been assessed to date in children with severe malaria. We hypothesized that children with severe malaria would have elevated plasma and CNS levels of sST2, which would correlate with markers of endothelial activation and neuronal damage and predict neurocognitive impairment. To test these hypotheses, we measured plasma and cerebrospinal fluid (CSF) sST2 levels in children with CM and plasma IL-33 and sST2 levels in children with CM or SMA, comparing them with biomarkers of endothelial activation and neuronal damage, and with neurocognitive scores, over a 2-year follow-up.

## METHODS

### Study Population.

This prospective study was performed at Mulago Hospital, Kampala, Uganda, from 2008 to 2015. Children with cerebral malaria (CM) or severe malarial anemia (SMA) were enrolled if they were between 18 months and 12 years of age. Occurrence of CM was defined as (1) coma (Blantyre Coma Score ≤2); (2) *Plasmodium falciparum* on blood smear; and (3) no other known cause of coma (eg, meningitis, a prolonged postictal state, or hypoglycemia-associated coma reversed by glucose infusion). Exclusion criteria for children with CM enrolled in this study included prior history of coma, head trauma, hospitalization for malnutrition, or cerebral palsy. Occurrence of SMA was defined as a positive *P. falciparum* blood smear and serum hemoglobin ≤5 mg/dL. Exclusion criteria for children with SMA enrolled in this study included impaired consciousness, seizures prior to admission, or other clinical evidence of CNS involvement. Asymptomatic children aged 18 months to 12 years were enrolled from the neighborhoods and extended households of those enrolled with severe malaria to act as community control children. Children with active or recent illness, chronic illness requiring medical care, or prior coma were excluded as community control children.

At enrollment, whole blood was collected from children with severe malaria and from community control children and stored at -80°C to be used for further testing. In children with CM, samples of CSF were obtained to rule out bacterial meningitis or encephalitis in all children whose parents agreed to the procedure and in whom the procedure was not contraindicated. We used CSF samples from North American children treated for prior leukemia, in whom CSF was obtained to rule out return of malignancy, and who had no evidence of CNS disease at collection to evaluate CSF levels of ST2 in a control group. Plasma from these individuals was not available. Children with severe malaria were treated at Mulago Hospital according to Ugandan national treatment guidelines at the time. Follow-up of enrolled patients was conducted at 6, 12, and 24 months after discharge.

### Clinical and Demographic Assessments.

All children underwent a medical history and physical examination at enrollment. Peripheral blood smears were assessed for *Plasmodium* species by microscopy with Giemsa staining using standard protocols. Nutritional status was assessed by height-for-age and weight-for-age z-scores (WHO Child Growth Standards), and socioeconomic status was measured using a validated scoring system published previously [32]. Duration of coma was defined as time from admission until the child regained full consciousness (Blantyre Coma Score=5 or Glasgow Coma Scale=15). Acute kidney injury was defined as a 1.5-fold increase in creatinine from estimated baseline as described [33], using the Kidney Disease: Improving Global Outcomes guidelines based on a single-admission creatinine level [34].

### Biomarker Assessments.

The following methods were used for testing: plasma and CSF soluble ST2 (DuoSet ELISA kit, R&D Systems, Minneapolis, MN), plasma diluted 1:20, CSF samples diluted 1:4), plasma IL-33 (Luminex assay, Luminex Corp, Austin, TX), plasma *Plasmodium falciparum* histidine-rich protein-2 (PfHRP-2), (Malaria Ag CELISA kit, Cellabs, Brookvale, Australia), plasma soluble intracellular adhesion molecule-1 (sICAM-1), soluble vascular adhesion molecule-1 (sVCAM-1), vascular endothelial growth factor (VEGF), sE-selectin, sP-selectin, as well as plasma and CSF IL-1b (magnetic cytometric bead assay, R&D Systems); plasma and CSF TNF-α and IL-6 (magnetic cytometric bead assay, Millipore Sigma, Burlington, MA); plasma angiopoietin-1 (Angpt-1) and -2 (Angpt-2) (DuoSet ELISA kit, R&D Systems); and plasma Von Willebrand Factor (vWF) (ELISA, Corgenix Medical Corp, Broomfield, CO). Plasma and CSF albumin were quantified by the Advanced Research and Diagnostic Laboratory at the University of Minnesota using the Bromocresol Purple Albumin Assay (Sigma-Aldrich, St. Louis, MO). The CSF/plasma albumin index ([CSF albumin concentration/plasma albumin concentration] x 1000) was used as a surrogate measure of blood-brain barrier damage. CSF tau testing was performed using the Luminex-based Human Tau (total) Singleplex Bead Kit (Invitrogen, Carlsbad, CA) and the Human Neuroscience Buffer Reagent Kit (Invitrogen). Plasma tau levels were measured via ultrasensitive biomarker detection at Quanterix (Billerica, MA).

### Neurologic and Cognitive Assessments.

Neurologic and cognitive assessments were performed at discharge (neurologic testing) or 1 week after discharge (cognitive testing), and at 6, 12, and 24 months after discharge. A neurologic deficit was defined as presence of motor or cranial nerve deficit, ataxia, a movement disorder, or clinically detectable behavioral, speech, or visual disorders. The tests used to assess cognition were validated in cohorts of Ugandan children and described in detail in prior publications [2, 6, 35]. Different testing batteries were used in children <5 years of age vs ≥5 years of age because no in-depth cognitive testing method is appropriate for the full age span. In brief, for children <5 years of age, cognitive ability was assessed by the Mullen Scales of Early Learning [36], attention by the Early Childhood Vigilance Test [37], and associative memory by the Color Object Association Test [38]. In children ≥5 years, overall cognitive ability was assessed by the summary mental processing index of the Kaufman Assessment Battery for Children, second edition (KABC-II) [39], attention by the Test of Variables of Attention (D prime measure primary outcome) [40], and working memory by the sequential processing subtest of the K-ABC-II (44). To account for differences in child age, we converted each raw score into a z-score using scores of the community control children. The z-scores were computed as (actual score-mean score for a child's age)/standard deviation (SD), where the mean score for a child's age and SD were computed by fitting a quadratic mixed effects model, including a random intercept for the child and where correlations within a child were based on time between visits, to data for all visits for all community control children.

### Statistical Analysis.

Analyses were conducted using Stata/SE14 (StataCorp, College Station, TX). For continuous variables, Wilcoxon Rank Sum tests were performed to compare differences between 2 groups, and Kruskal-Wallis tests were used to compare differences among all 3 groups. Categorical or ordinal variables, such as sex and preschool education, were analyzed using Chi-Squared tests. Spearman's correlation was used to assess the association between plasma and CSF levels of sST2. Linear, logistic, and negative binomial regression analyses were used to compare sST2 levels (log10-transformed) to continuous, dichotomous, and count outcomes, respectively. These comparisons were corrected for multiplicity using the Bonferroni correction.

For cognitive outcomes, linear mixed-effects models were used to allow for comparison of plasma and CSF sST2 concentrations to test overall testing time points in the 2-year follow-up. The models were adjusted for factors that could impact cognitive outcomes, including age, sex, height-for-age z-score, weight-for-age z-score, plasma levels of HRP2, and preschool education of study participants. In the models, within-subject observations were correlated using a subject-specific intercept and timepoints were treated as categorical variables. A banded diagonal covariance matrix was assumed to model within-subject variance-covariance errors, the mixed models were fitted by restricted maximum likelihood, and Kenward-Roger approximations were used to estimate the denominator degrees of freedom.

### Ethical Review.

Written informed consent was obtained from parents or guardians of study participants. Ethical approval was granted by the institutional review boards for human studies at the Makerere University School of Medicine, the Uganda National Council for Science and Technology, and the University of Minnesota Medical School.

## RESULTS

### Study cohort demographic characteristics.

**Figure 1.**
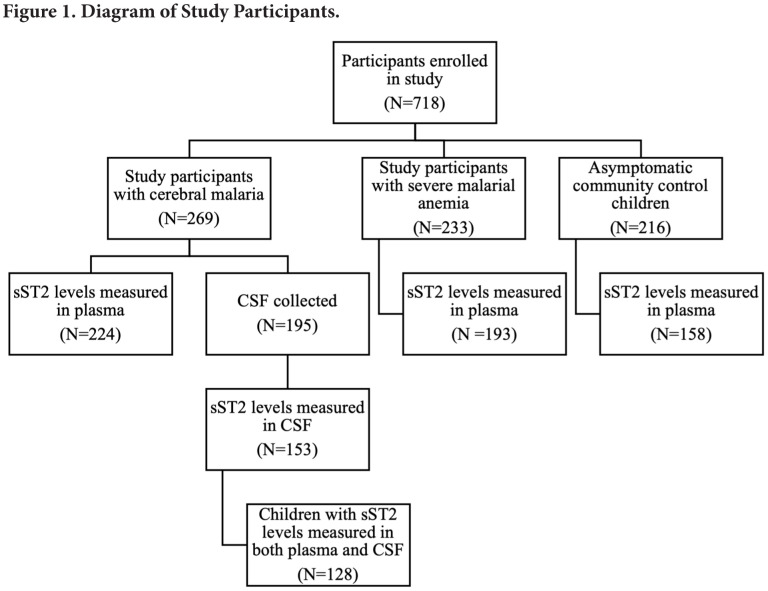
Loss of sample size generally caused by insufficient volume of plasma or CSF samples for testing. Abbreviations: CSF, cerebrospinal fluid

Soluble ST2 (sST2) was measured in the plasma of 224 children with CM, 194 children with SMA, and 158 asymptomatic community control children (Figure 1). Of the 269 children enrolled with CM, samples of CSF were collected in 195 children; sST2 was measured in 153 children due to limited sample volume (Figure 1). Measurement of both plasma and CSF levels of sST2 was performed in 128 of the children with CM (Figure 1).

Children with CM and community control children were older than children with SMA (median age, years [interquartile range] CM 3.49 [2.48, 4.88], community controls 3.50 [2.63, 4.61], SMA 2.79 [2.04, 4.35], *P*<0.001), and there was a greater proportion of male children in the CM and SMA groups compared with community control children (male %, CM, 59.4%, SMA, 60.1%, community control, 44.9%, *P*=0.006). Mean [standard deviation] days of fever prior to admission did not differ significantly between children <5 years old and children ≥ 5 years old among children with CM (3.53 [1.99] vs 3.20 [1.47], *P*=0.14) or SMA (4.13 [2.84] vs 4.48 [3.34], *P*=0.53).

### Plasma IL-33 is rarely detected in children with severe malaria or in community children.

IL-33 was tested for in 405 plasma samples of children with CM (n=170), children with SMA (n=169), or community control children (n=66). Levels were below the limit of detection (2.67 ng/ml) in 362 samples (89.4%), so we did not test IL-33 in further samples. IL-33 was not measured in CSF due to limited CSF sample volume. Among study participants with non-zero values for IL-33 in plasma, there was no significant difference between study groups (*P*>0.05 for all comparisons).

### Plasma sST2 levels are elevated in children with CM and children with SMA compared with community controls.

**Figure 2.**
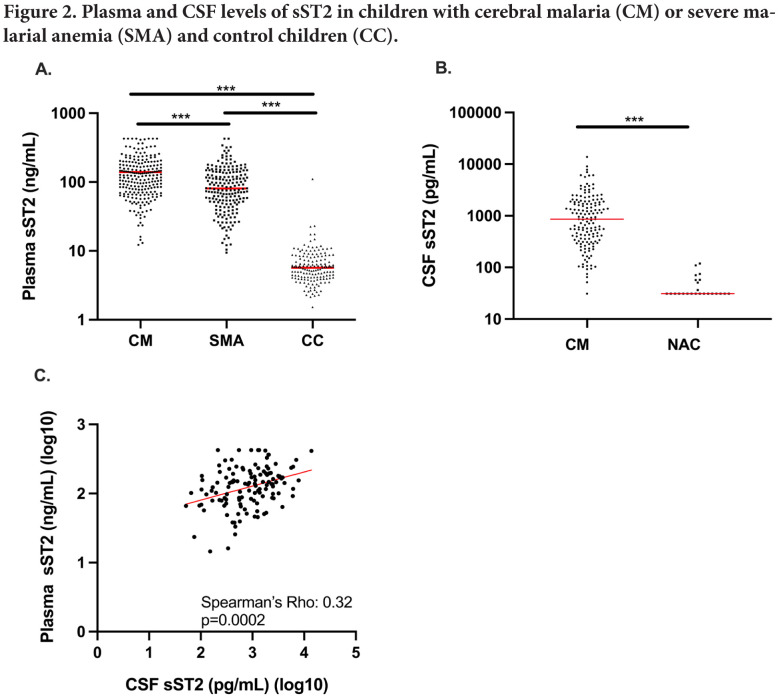
Plasma and CSF levels of sST2 in Ugandan children with cerebral malaria, children with severe malarial anemia, and community control children. (A) Levels of sST2 were elevated in children with CM (n=224) or SMA (n=193) compared with asymptomatic community control children (CC, n=156). (B) Concentration of sST2 in the CSF of children with CM (n=153) was higher compared to that of healthy North American Control (NAC) children (n=24). (A,B) Wilcoxon Rank Sum Test was used to compare each group. ***P<0.0001. (Circles) Plasma sST2 concentration in individual children, (horizonal bar) median sST2 concentration for each group. (C) Plasma and CSF levels of sST2 were correlated using Spearman's correlation (rho=0.32, *P*=0.0002, n=128). (Red line) Line of best fit based on linear regression model. Abbreviations: CM, cerebral malaria; CSF, cerebrospinal fluid; SMA, severe malarial anemia; CC, control children

Plasma sST2 levels were higher in children with CM (median (ng/mL) [interquartile range: IQR] 116.7 [70.8-178.5]) than in children with SMA (81.0 [50.2-139.4]), and both were higher than levels in community control children (5.7 [4.1-8.2]) (all *P*<0.0001; Figure 2A). Plasma sST2 levels did not differ by age group in CM or SMA (all *P*>0.05) or by duration of fever (Spearman's *rho* = -0.068, *P*=0.17), so appeared to reflect severity of disease rather than duration of symptoms.

### CSF sST2 levels are elevated in children with CM and correlate with plasma sST2 levels.

CSF sST2 levels were significantly higher in children with CM than in control samples from North American children (median (pg/mL) [IQR], CM, 860.8 [342.5-1826.2]; North American children, 31.2, [31.2, 35.1]. *P*<0.0001; Figure 2B). CSF sST2 levels correlated positively with plasma sST2 levels in children with CM (Spearman's rho, 0.32, *P*=0.0002, n=128; Figure 2C). The CSF/plasma albumin index, a marker of blood-brain barrier damage, correlated with CSF sST2 levels (beta coefficient, 95% CI, 0.71 [0.47,0.95], *P*<0.001; Table 1). Plasma and CSF sST2 levels did not differ according to presence or absence of malaria retinopathy in children with CM (median [IQR] for plasma, 122.3 [78.4-179.9] vs 114 [63.3-175.8], *P*=0.38; CSF, 720.0 [310.6-1806.5] vs 1093.8 [460.8-1826.2], *P*=0.44, respectively).

**Table 1.** Associations between plasma and CSF levels of sST2 and parasite and host response factors in children with cerebral malaria (CM) or severe malarial anemia (SMA)

	CM CSF ST2 level			CM plasma ST2 level			SMA plasma ST2 level		
	n	beta coefficient [95% CI]	*P* value	n	beta coefficient [95% CI]	*P* value	n	beta coefficient [95% CI]	*P* value
**Parasite burden**									
Peripheral blood parasite density	147	−0.01 [−0.09, 0.07]	0.81	218	0.05 [0.01, 0.09]	**0.02**	193	0.03 [−0.02, 0.07]	0.29
Plasma *Pf*HRP-2 level	153	0.21 [0.08, 0.35]	**0.003**	224	0.14 [0.07, 0.20]	**<0.001**	193	0.08 [0.02, 0.14]	**0.009**
**Plasma markers of endothelial activation**									
sICAM-1	140	−0.10 [−0.24, 0.04]	0.17	172	0.12 [0.05, 0.20]	**0.002**	157	0.07 [−0.00, 0.14]	0.06
sVCAM-1	140	0.68 [0.37, 0.99]	**<0.001**	172	0.34 [0.16, 0.52]	**<0.001**	157	0.50 [0.29, 0.70]	**<0.001**
Angiopoietin 1	135	−0.19 [−0.35, -0.02]	0.02	189	−0.07 [−0.15, 0.01]	0.07	154	−0.11 [−0.22, -0.01]	0.03
Angiopoietin 2	135	0.16 [−0.05, 0.37]	0.13	189	0.27 [0.17, 0.37]	**<0.001**	154	0.26 [0.16, 0.36]	**<0.001**
Ratio Angpt1: Angpt2	135	0.20 [0.07, 0.34]	**0.003**	189	0.15 [0.09, 0.21]	**<0.001**	154	0.21 [0.13, 0.28]	**<0.001**
VEGF	140	0.10 [−0.10, 0.30]	0.32	195	−0.06 [−0.16, 0.04]	0.24	157	−0.09 [−0.23, 0.05]	0.21
PDGF	140	−0.15 [−0.34, 0.04]	0.12	195	−0.11 [−0.20, -0.02]	0.02	157	−0.06 [−0.17, 0.06]	0.32
E-selectin	140	0.33 [−0.10, 0.75]	0.13	172	0.61 [0.39, 0.84]	**<0.001**	157	0.59 [0.35, 0.82]	**<0.001**
P-selectin	136	−0.03 [−0.41, 0.34]	0.86	164	0.55 [0.34, 0.76]	**<0.001**	90	0.27 [0.01, 0.54]	0.04
Von Willebrand Factor	124	−0.14 [−0.42, 0.14]	0.33	157	0.12 [−0.02, 0.27]	0.10	135	0.20 [0.04, 0.36]	0.02
**Markers of neuronal injury**									
Plasma tau level	106	0.15 [−0.07, 0.38]	0.18	177	0.16 [0.06, 0.27]	**0.003**	153	0.32 [0.18, 0.47]	**<0.001**
CSF tau level	122	0.12 [−0.08, 0.32]	0.24	114	0.21 [0.09, 0.33]	**<0.001**			
CSF:plasma albumin index	131	0.71 [0.47, 0.95]	**<0.001**	121	0.18 [0.02, 0.35]	0.03			

All factors are continuous variables and were log transformed (base 10) prior to linear regression analysis. Data presented as beta coefficient and 95% CI. Corrected for multiple comparisons using Bonferroni correction within each type of marker, *P*<0.025 for parasite factors, *P*<0.005 for markers of endothelial activation, and *P*<0.025 for markers of neuronal injury.

Abbreviations: CM, cerebral malaria; CSF, cerebrospinal fluid; SMA, severe malarial anemia; CI, confidence interval; *Pf*HRP-2*, P. falciparum* histidine-rich protein; Ratio Angpt 1:Angpt 2, ratio of plasma levels of angiopoietin 1 to angiopoietin 2. See Methods for abbreviations of additional markers of endothelial activation.

### Plasma and CSF levels of sST2 correlate with parasite biomass and endothelial activation in children with CM.

Total parasite biomass, as assessed by plasma *Pf*HRP2 level, correlated positively with plasma sST2 levels in children with CM or SMA and correlated positively with CSF sST2 levels in children with CM (Table 1). Peripheral blood parasite density correlated positively with plasma sST2 level in children with CM but not in children with SMA (Table 1).

Plasma levels of sST2 in children with CM and in children with SMA were associated with increased levels of multiple markers of endothelial activation, including sVCAM-1, Angpt-2, the ratio of Angpt-2 to Angpt-1, E-selectin, and P-selectin (Table 1). Among these markers, sVCAM-1 and the ratio of Angpt-2 to Angpt-1 were associated with increased CSF concentrations of sST2 in children with CM (Table 1).

### Plasma levels of sST2 correlate with plasma and CSF tau levels in children with CM.

Plasma sST2 levels correlated strongly with both plasma and CSF levels of tau, a marker of neuronal injury, in children with CM, and with plasma tau levels in children with SMA (Table 1).

### Plasma sST2 levels correlate with plasma TNF-α levels in CM and SMA, while CSF sST2 levels correlate with CSF IL-6 and TNF-α levels in CM.

In ECM, absence of the ST2/IL-33 pathways was associated with decreased expression of the pro-inflammatory cytokines IL-1b, TNF-α, and IL-6 in the brain [27, 28]. For this reason, we compared these levels in children with CM or SMA. Plasma sST2 levels in children with CM or SMA were associated with increased plasma levels of the pro-inflammatory cytokine TNF-α (Table 2). In children with CM, where CSF levels of these cytokines could be measured, CSF sST2 levels were positively associated with CSF levels of IL-6 and TNF-α (Table 2). Plasma and CSF sST2 levels were not associated with either plasma or CSF IL-1b levels in children with CM (Table 2).

**Table 2.** Association between plasma and CSF levels of sST2 to plasma and CSF pro-inflammatory cytokine levels.

	CM CSF ST2 level			CM plasma ST2 level			SMA plasma ST2 level		
	n	beta coefficient [95% CI]	*P* value	n	beta coefficient [95% CI]	*P* value	n	beta coefficient [95% CI]	*P* value
**Cytokines in Plasma**									
IL-6	142	−0.04 [−0.13, 0.05]	0.42	206	0.06 [0.01, 0.10]	0.02	183	0.18 [0.11, 0.25]	**<0.001**
TNF-α	142	0.01 [−0.12, 0.15]	0.84	206	0.13 [0.05, 0.20]	**0.001**	183	0.20 [0.09, 0.31]	**<0.001**
IL-1b	140	0.15 [−0.06, 0.35]	0.16	195	−0.03 [−0.14, 0.08]	0.57	157	−0.03 [−0.15, 0.09]	0.62
**Cytokines in CSF**									
IL-6	153	0.24 [0.14, 0.35]	**<0.001**	147	0.02 [−0.04, 0.09]	0.49			
TNF-α	153	0.30 [0.22, 0.39]	**<0.001**	147	0.05 [−0.01, 0.11]	0.14			
IL-1b	122	0.10 [−0.14, 0.35]	0.40	114	0.09 [−0.10, 0.28]	0.35			

All factors are continuous variables and were log transformed (base 10) prior to linear regression analysis. Data presented as beta coefficient and 95% CI. Corrected for multiple comparisons using Bonferroni correction within each type of marker, *P*<0.013 for cytokines in plasma and CSF.

Abbreviations: CM, cerebral malaria; CSF, cerebrospinal fluid; SMA, severe malarial anemia; CI, confidence interval.

### Plasma sST2 levels are associated with acute kidney injury and thrombocytopenia but not mortality in children with CM.

To evaluate the clinical significance of elevated plasma and CSF sST2 levels, we compared them to mortality in children with CM, and to risk of acute kidney injury, a major complication of CM and SMA, and thrombocytopenia, which occurs more often in children with severe malaria than uncomplicated malaria. Plasma and CSF sST2 levels were not associated with mortality in children with CM (Supplementary Table 1). However, plasma sST2 levels were associated with an increased risk of acute kidney injury and elevated blood urea nitrogen in children with CM and in children with SMA (Table 3), and with a greater risk of thrombocytopenia, another marker of disease severity, in children with CM and in children with SMA (Table 3).

**Table 3.** Association between plasma and CSF levels of sST2 to clinical risk factors in children with cerebral malaria (CM) or severe malarial anemia (SMA)

	CM CSF			CM Plasma			SMA Plasma		
	n,N^a^	OR [95% CI]	*P* value	n,N^a^	OR [95% CI]	*P* value	n,N^a^	OR [95% CI]	*P* value
**Clinical Risk Factorsb**									
Lactic acidosis	46, 145	1.32 [0.66, 2.63]	0.43	66, 207	3.23 [1.19, 8.77]	0.02	80, 178	1.57 [0.63, 3.93]	0.33
Acute kidney injury	67, 149	1.81 [0.94, 3.50]	0.08	88, 215	7.61 [2.73, 21.21]	**<0.001**	44, 187	10.50 [2.98, 37.04]	**<0.001**
Elevated BUN	65, 153	2.38 [1.21, 4.68]	**0.01**	90, 224	43.13 [12.25, 151.87]	**<0.001**	52, 193	63.66 [13.95, 290.64]	**<0.001**
Thrombocytopenia	133, 153	2.73 [1.06, 7.08]	0.04	190, 219	7.77 [2.08, 29.05]	**0.002**	100, 192	3.52 [1.42, 8.71]	**0.007**

Associations among levels of sST2 in children with severe malaria with clinical risk factors and clinical lab tests. Plasma and CSF levels of sST2 were log transformed (base 10). Odds ratios (OR) and 95% confidence intervals (CI) are presented. Corrected for multiple comparisons using Bonferroni correction, *P*<0.013.

Definitions: lactic acidosis, lactate >5 nmol/L, uremia, plasma urea nitrogen >20 mg/dL; thrombocytopenia, platelet count <150,000.

a n,N denotes the number of children included in the analysis that were positive for each clinical risk factor compared to the total number of children included in the analysis.

b Clinical risk factors are binary outcomes and were analyzed using logistic regression models.

Abbreviations: CM, cerebral malaria; CSF, cerebrospinal fluid; SMA, severe malarial anemia; OR, odds ratio; CI, confidence interval; BUN, blood urea nitrogen.

### Elevated levels of sST2 in plasma are associated with long-term neurocognitive impairment in children with CM.

**Figure 3.**
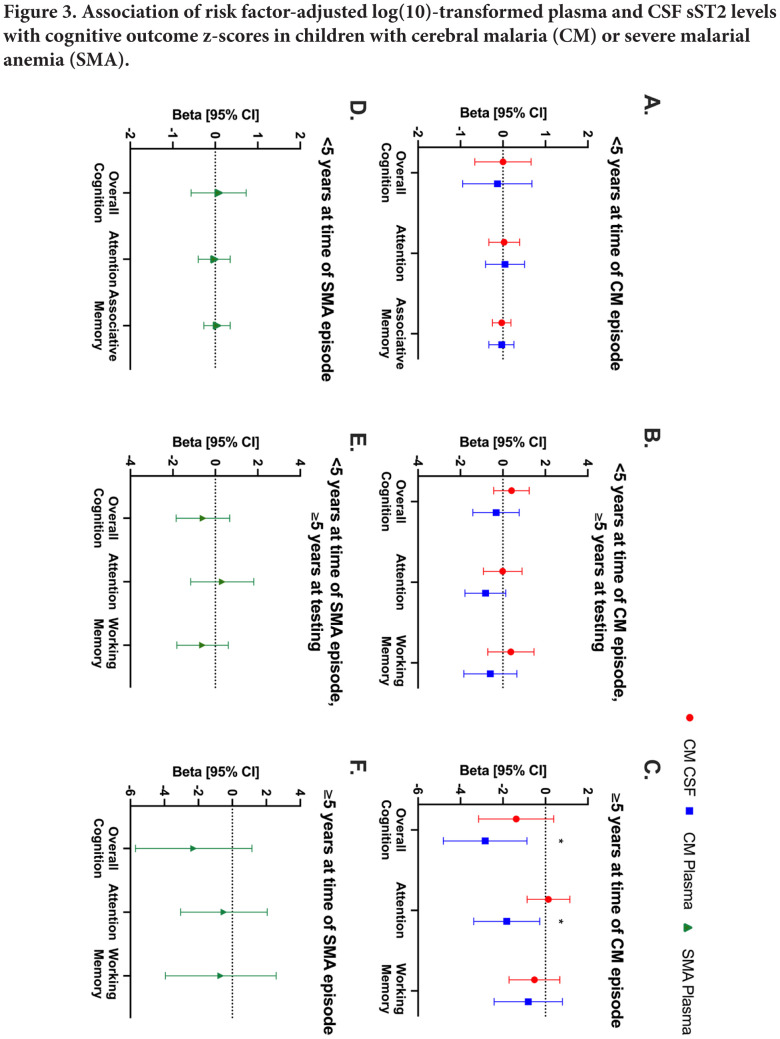
Associations of plasma and CSF sST2 concentration with cognitive outcomes in children with cerebral malaria or severe malarial anemia. Associations between CSF and plasma levels of sST2 (log10 transformed) with cognitive outcomes in children with CM <5 years old at the time of CM episode (A) and throughout the 24-month follow-up period, children who turned 5 years old during follow-up and underwent cognitive testing using tests for children over 5 years old (B), and children who were over 5 years old at time of CM episode and throughout follow-up (C). (D–F) Associations between CSF and plasma levels of sST2 (log10 transformed) with cognitive outcomes in children with SMA for the same age groups. Data are presented as beta coefficient and 95% confidence interval from a linear mixed effects model. (Red) sST2 concentration in CSF from children with CM, (blue) sST2 concentration in plasma from children with CM, (green) sST2 concentration in plasma from children with SMA. The model is adjusted for age, sex, height-for-age z-score, weight-for-age z-score, plasma levels of HRP2, and preschool education of study participants. See Supplementary Table 1 for beta coefficients and 95% confidence intervals for unadjusted analysis and sample sizes. *P<0.05. Abbreviations: CM, cerebral malaria; CSF, cerebrospinal fluid; SMA, severe malarial anemia; CI, confidence interval

In children with CM, there was no significant association between plasma or CSF sST2 levels and neurological deficits at any time point. Children with neurologic deficits at 24 months had greatly increased odds of elevated plasma sST2 levels at admission, but with wide confidence intervals (Supplementary Table 1). Admission plasma sST2 levels could be predictive of the most persistent neurologic deficits, but further studies with larger numbers will be required to confirm this. In children with CM who were <5 years of age at the time of their CM episode and at the time of cognitive testing, or who were <5 years of age at the time of CM episode but ≥5 years at testing, risk factor-adjusted plasma or CSF sST2 levels were not significantly associated with any cognitive outcome (Figure 3A,B). However, in children with CM who were ≥5 years at the time of the CM episode, risk factor-adjusted plasma sST2 concentrations were associated with worse overall cognitive ability (beta coefficient [95% confidence interval]; –2.84 [–4.81, –0.87] (n=46), *P*=0.01) and attention (–1.82 [–3.39, –0.27], *P*=0.02; Figure 3A-C; Supplementary Table 2).

In children with SMA, there were no significant associations in unadjusted or adjusted models with any cognitive outcome in any age group (Figure 3D-F; Supplementary Table 2). Children with CM or SMA who had plasma or CSF sST2 levels tested did not differ significantly from those who did not have plasma or CSF sST2 testing in age, sex, or cognitive outcome scores (Supplementary Table 3).

## DISCUSSION

In the present study, we provide the first evidence that sST2 is a marker of disease severity in children with severe malaria and that elevated plasma sST2 levels predict long-term cognitive impairment in children ≥5 years of age with cerebral malaria (CM). The study associations suggest a potential pathway for neuronal injury: activation of the IL-33/ST2 pathway by increased parasite biomass in children with CM; leading to release of sST2, release of pro-inflammatory cytokines, endothelial activation, and blood-brain barrier dysfunction; which in turn contribute to acute neuronal injury and long-term cognitive impairment. Our study shows association of admission plasma sST2 levels with neuronal injury and cognitive impairment, and the pathway outlined is consistent with experimental cerebral malaria (ECM) studies in which the IL-33/ST2 pathway initiated oligodendrocyte and microglial responses in early infection, resulting in greater neuroinflammation, neuronal damage, and associated neurological and cognitive deficits [28].

Endogenous and exogenous IL-33 appear to have differing effects in murine models of severe malaria. Endogenous IL-33 leads to an increase in Th1 responses [28, 41], while exogenous IL-33 induces a Th2 response through induction of innate lymphoid cells, M2 macrophages, and regulatory T cells [29], and through inhibition of the NLP3 inflammasome [30]. In murine ECM studies, exogenous IL-33 prevented ECM if given early [29] and reduced mortality and neuro-cognitive effects of ECM if given at the first sign of neurologic deficit [30]. These studies suggest that exogenous IL-33 could be of benefit as adjunctive therapy early in cerebral malaria. The only prior study of IL-33 in human malaria showed high plasma levels of IL-33 in children with severe malaria [42]. In the present study, few children had detectable IL-33 in plasma. The association of elevated plasma sST2 levels with endothelial activation, inflammation, neuronal injury, and long-term neurocognitive impairment in children with CM supports further investigation of exogenous IL-33 or IL-33 analogs to suppress the pro-inflammatory response elicited by sST2 in murine or non-human primate models, to evaluate how the timing and dose of administration impact disease outcomes and host response in a model. Further research is required before these agents could be considered for human studies.

A key strength of the present study was the measurement of levels of sST2 and cytokines in CSF, providing a measure of CNS sST2 activity. Our findings revealed that CSF sST2 levels are elevated, that much of this elevation is likely due to plasma sST2 crossing the impaired blood-brain barrier, and that direct effects of CSF sST2 do not appear to be a major component in CNS neuronal injury, since plasma but not CSF sST2 levels correlated with CSF tau levels. The association of plasma sST2 levels with levels of plasma tau in children with CM [43] was of particular interest, because it provides a potential pathway through which IL-33/sST2 might lead to long-term neurocognitive impairment. A recent study in this same study cohort demonstrated that plasma tau concentrations were associated with cognitive deficits in children with CM but not SMA [44], suggesting that long-term neurocognitive impairment may be occurring by a different mechanism in children with CM than SMA. Similarly, in the current study, plasma sST2 levels in children with CM but not in children with SMA were associated with worse cognitive outcomes, despite children in both severe malaria groups demonstrating elevated levels of plasma sST2, suggesting that the ST2 pathway may be more important in brain injury in CM compared to SMA.

CSF sST2 levels correlated with levels of CSF pro-inflammatory (IL-6, TNF-α) cytokines, suggesting that IL-33/sST2 may contribute to neuronal injury indirectly, through induction of inflammation. In addition, plasma sST2 correlated with plasma TNF-α, which can cause direct and indirect neuronal injury through ischemia [45], spontaneous demyelination [46], and induction of the deaths of neurons and oligodendrocytes [47]. Plasma sST2 also correlated with an elevated angiopoietin-2/angiopoietin-1 ratio, which has been seen in and is thought to contribute to sequelae from traumatic brain injury [48]. It is not clear whether sST2 affects endothelial activation, endothelial activation affects sST2 levels, or if interactions occur both ways. Animal studies or studies of an in vitro blood-brain barrier could help to determine this [49]. Plasma sST2 levels may also, or instead, serve as a marker of brain injury, elevated as a specific response to cerebral ischemia and serving as a marker of neuroinflammation [19]. The link between plasma sST2 and tau in this study, along with the associations with cognitive impairment in this population, and similar findings in an ECM model with IL-33 upregulation, suggest a connection between the ST2/IL-33 pathway and neuronal damage.

Elevated plasma sST2 levels were associated with worse cognitive outcome scores only in children with CM ≥5 years of age. The lack of association of plasma sST2 in children who were <5 years at CM episode and ≥5 years at time of testing suggests that this association was not due solely to effects of plasma sST2 elevation on cognitive pathways only measurable once the child is 5 years of age or older. Instead, the role of IL-33/sST2 in cognitive impairment may be greater in older children with less neural plasticity. In our study on plasma tau levels in this cohort, we found the opposite: plasma tau levels were associated with cognitive impairment only in children with CM <5 years of age. The contrasting findings raise the possibility that axonal neuronal injury, as assessed by plasma tau levels, may be more important in long-term cognitive impairment in children <5 years of age, while injury of other brain cells may predominate in children ≥5 years of age. Brain injury biomarkers that evaluate injury to other brain cell types should be evaluated in this population. Similarly, plasma sST2 and the activity it represents in the ST2/IL-33 pathway, may be more important in the pathways leading to cognitive impairment in children ≥ 5 years of age. The association with neurocognitive impairment in children in CM but not SMA suggests either that this process is more important in CM or that the sST2 levels seen in CM, which were significantly higher than those in SMA, are needed to cause long-term brain injury.

Elevated plasma levels of sST2 were associated not only with inflammation and neurocognitive impairment in children with CM, but also with clinical findings associated with development of severe malaria (thrombocytopenia [50]) or with mortality in severe malaria (acute kidney injury [33, 51], and uremia [52, 53]). In acute kidney injury, the roles of IL-33 and sST2 are even less clear: in a study of myocardial infarction, elevated sST2 predicted acute kidney injury [54], but murine studies have shown IL-33-dependent endothelial activation, a process that should be dampened by elevated sST2 levels, leading to acute kidney injury after infection [55]. Additionally, in mice, exogenous IL-33 leads to further kidney damage, but exogenous sST2 leads to renal protection in mice after cisplatin-induced acute kidney injury [56]. In the present study, elevated sST2 levels were strongly associated with acute kidney injury in children with CM or SMA, suggesting a potential role for sST2 in malaria-associated acute kidney injury through endothelial activation.

Study strengths include a large sample size for a study of this kind; inclusion of community controls; longitudinal follow-up of study participants; rigorous assessment of clinical complications and assessment of multiple pathways implicated in severe malaria pathogenesis; evaluation of key biological factors in the potential disease pathway, including measures of parasite biomass, endothelial activation, inflammation, and neuronal injury; and in-depth evaluation of long-term cognitive outcomes. A study limitation is that, as in all human studies, pathways could not be manipulated to determine directly whether changes in the ST2 pathway resulted in different clinical or neurocognitive outcomes. In addition, sST2 levels were measured at a single timepoint (hospital admission), rather than over multiple timepoints before and after admission. However, this measurement is probably the most relevant for interventions, which would be given at the time of admission for severe disease. In a study of serum sST2 levels in adults with sepsis, sST2 levels were highest 1 day after onset of disease but remained elevated for at least 7 days after the episode [21]. Evaluation of the kinetics of plasma or serum sST2 levels during severe malaria would be of interest to see how they compare in contrast.

In conclusion, we found that plasma sST2 levels are elevated in children with CM and in children with SMA, with the greatest elevation seen in children with CM; that CSF sST2 levels are also elevated in children with CM, likely due to leakage of plasma sST2 across an impaired blood-brain barrier; that elevated plasma sST2 is associated with an increase in parasite biomass, endothelial activation, and levels of the neuronal injury marker tau; and that in children with CM who are ≥5 years of age at the time of CM, elevated plasma sST2 is associated with long-term neurocognitive impairment. Together, the study findings suggest that sST2 may be a useful marker for disease severity in malaria, and in conjunction with prior ECM studies suggest a potential role for sST2 in induction of endothelial activation, neuroinflammation, neuronal injury, and long-term neurocognitive impairment in children with CM. Further research will be required to determine if factors that affect the ST2 pathway may be used safely as adjunctive treatment in severe malaria.

## SUPPLEMENTARY DATA

Supplementary materials are available at the Pathogens and Immunity website. Supplementary data may be provided by the authors to benefit the reader. Supplementary data are not copyedited and are the sole responsibility of the authors. Questions or comments related to supplementary materials should be addressed to the corresponding author.
