# Supplementary material for: Elevated Plasma Soluble ST2 Levels are Associated With Neuronal Injury and Neurocognitive Impairment in Children With Cerebral Malaria
Source: Pathog Immun. 2022 Jun 23;7(1):60–80. doi: 10.20411/pai.v7i1.499 (PMC9254869; doi:10.20411/pai.v7i1.499)
Supplement: Supplementary Tables 1-3 [file pai-7-060-s01.pdf]

PATHOGENS AND IMMUNITY
IN ASSOCIATION WITH CASE WESTERN RESERVE UNIVERSITY AND THE RICHARD J. FASENMYER FOUNDATION

SUPPLEMENTARY TABLES

Supplemental Table 1. Associations of admission log(10)-transformed plasma and CSF levels of sST2 with in-hospital outcomes and neurologic deficits in children with CM.

		CM plasma			CM CSF		
**Clinical outcome**	**Value**	**n/N[a]**	**OR, beta, IRR [95% CI]**	***P* value**	**N[a]**	**OR, beta, IRR [95% CI]**	***P* value**
Mortality	OR	29/224	1.56 [0.42, 5.80]	0.51	9/153	3.57 [0.82, 15.62]	0.09
Coma duration[b]	ß	195	0.13 [-0.02, 0.29]	0.10	144	0.03 [-0.07, 0.14]	0.55
No. of seizures in hospital	IRR	224	0.67 [0.09, 1.24]	**0.02**	153	-0.10 [-0.52, 0.33]	0.66
**Presence of neurological deficits at:**							
Discharge	OR	69/190	0.66 [0.25, 1.73]	0.40	57/143	1.11 [0.56, 2.20]	0.76
6-month follow-up	OR	10/186	3.27 [0.37, 28.56]	0.28	8/139	1.66 [0.38, 7.35]	0.50
12-month follow-up	OR	7/185	8.28 [0.56, 123.0]	0.13	5/139	2.31 [0.35, 15.35]	0.29
24-month follow-up	OR	6/183	16.80 [0.82, 344.93]	0.07	4/137	2.31 [0.28, 19.0]	0.44

Corrected for multiple comparisons using Bonferroni correction, $P<0.017$ for clinical outcome markers and $P<0.013$ for presence of neurological deficits.

[a]n/N for dichotomous outcomes (mortality and presence of neurological deficits) denotes number of children who died (mortality) or had neurological deficits (n) at each time point, over the total number of children tested/evaluated at that time point (N)
[b]Coma duration log-transformed (base 10)

Abbreviations: CM, cerebral malaria; CSF, cerebrospinal fluid; OR, odds ratio; IRR, incidence rate ratio; CI, confidence interval.

Fernander EM, Adogamhe P, Datta D, Bond C, Zhao Y, Bangirana P, Conroy AL, Opoka RO, John CC. Elevated plasma soluble ST2 levels are associated with neuronal injury and neurocognitive impairment in children with cerebral malaria. *Pathogens and Immunity*. 2022;7(1): 60–80. doi: 10.20411/pai.v7i1.499

PATHOGENS AND IMMUNITY
In Association with Case Western Reserve University and The Richard J. Fasenmyer Foundation

Supplemental Table 2. Plasma and CSF sST2 concentration associations with cognitive outcomes in children with cerebral malaria (CM) and severe malarial anemia (SMA).

	N(obs), n[a]	Unadjusted beta [95% CI]	Unadjusted *P* value	N(obs), n[a]	Adjusted beta[c] [95% CI]	Adjusted *P* value
**CSF sST2 levels compared to cognitive outcomes in children with CM**						
**<5 years at CM episode**						
Overall Cognition	336, 114	0.15 [-0.49, 0.79]	0.65	334, 112	0.00 [-0.66, 0.66]	1.00
Attention	355, 114	0.16 [-0.18, 0.50]	0.35	353, 112	0.03 [-0.33, 0.39]	0.88
Associative Memory	355, 115	0.07 [-0.14, 0.28]	0.49	353, 113	-0.03 [-0.25, 0.19]	0.76
**<5 years at CM episode; ≥5 years at time of testing**						
Overall Cognition	77, 57	**0.93 [0.02, 1.83]**	**0.04**	77, 57	0.41 [-0.43, 1.24]	0.33
Attention	79, 57	0.69 [-0.28, 1.65]	0.16	79, 57	-0.01 [-0.92, 0.90]	0.99
Working Memory	79, 57	0.98 [-0.15, 2.11]	0.09	79, 57	0.38 [-0.71, 1.47]	0.49
**≥5 years at time of CM episode**						
Overall Cognition	111, 29	**-1.68 [-3.03, -0.33]**	**0.02**	107, 28	-1.38 [-3.15, 0.39]	0.12
Attention	111, 29	-0.20 [-1.07, 0.66]	0.63	107, 28	0.14 [-0.86, 1.15]	0.77
Working Memory	112, 29	-0.89 [-1.90, 0.12]	0.08	108, 28	-0.52 [-1.72, 0.68]	0.37
**Plasma sST2 levels compared to cognitive outcomes in children with CM**						
**<5 years at CM episode**						
Overall Cognition	423, 143	0.08 [-0.80, 0.95]	0.86	421, 141	-0.13 [-0.95, 0.68]	0.75
Attention	443, 144	0.15 [-0.30, 0.59]	0.51	441, 142	0.05 [-0.41, 0.51]	0.84
Associative Memory	440, 144	-0.01 [-0.30, 0.29]	0.97	438, 142	-0.03 [-0.33, 0.26]	0.82
**<5 years at CM episode; ≥5 years at time of testing**						
Overall Cognition	105, 76	-0.28 [-1.62, 1.05]	0.67	105, 76	-0.32 [-1.42, 0.77]	0.56
Attention	106, 76	-0.66 [-1.75, 0.43]	0.23	106, 76	-0.82 [-1.78, 0.14]	0.09
Working Memory	106, 76	-0.38 [-1.69, 0.93]	0.57	106, 76	-0.59 [-1.84, 0.66]	0.35
**≥5 years at time of CM episode**						
Overall Cognition	188, 49	**-2.55 [-4.47, -0.62]**	**0.01**	179, 46	**-2.84 [-4.81, -0.87]**	**0.01**
Attention	186, 49	-1.35 [-2.77, 0.07]	0.06	177, 46	**-1.82 [-3.38, -0.27]**	**0.02**
Working Memory	189, 49	-0.75 [-2.29, 0.78]	0.33	180, 46	-0.81 [-2.42, 0.80]	0.32
**Plasma sST2 levels compared to cognitive outcomes in children with SMA**						
**<5 years at SMA episode**						
Overall Cognition	473, 156	0.11 [-0.57, 0.80]	0.75	464, 153	0.08 [-0.57, 0.73]	0.81
Attention	509, 157	-0.06 [-0.42, 0.30]	0.75	500, 154	-0.02 [-0.40, 0.35]	0.90
Associative Memory	489, 154	-0.05 [-0.35, 0.25]	0.73	480, 151	0.04 [-0.27, 0.35]	0.80

Fernander EM, Adogamhe P, Datta D, Bond C, Zhao Y, Bangirana P, Conroy AL, Opoka RO, John CC. Elevated plasma soluble ST2 levels are associated with neuronal injury and neurocognitive impairment in children with cerebral malaria. *Pathogens and Immunity*. 2022;7(1): 60–80. doi: 10.20411/pai.v7i1.499

**<5 years at SMA episode; ≥5 years at time of testing**						
Overall Cognition	78, 50	-0.57 [-2.00, 0.86]	0.43	78, 50	-0.58 [-1.84, 0.68]	0.36
Attention	81, 53	0.06 [-1.26, 1.38]	0.93	81, 53	0.33 [-1.16, 1.81]	0.66
Working Memory	81, 53	-0.55 [-1.74, 0.63]	0.35	81, 53	-0.60 [-1.81, 0.61]	0.32
**≥5 years at time of SMA episode**						
Overall Cognition	128, 34	-1.02 [-3.21, 1.18]	0.35	120, 32	-2.28 [-5.70, 1.15]	0.18
Attention	129, 34	0.33 [-1.26, 1.93]	0.67	122, 32	-0.50 [-3.05, 2.05]	0.69
Working Memory	132, 34	-0.49 [-2.40, 1.42]	0.61	124, 32	-0.68 [-3.94, 2.58]	0.67

Linear mixed effects model. Cognitive testing conducted at discharge, 6, 12, and 24 months. Data are presented as beta coefficient and 95% confidence interval.

[a]N(obs) represents number of events; N represents the number of participants. sST2 levels log10 transformed. Decrease number in adjusted model on account of missing data in adjusting variables.
[c]Model adjusted for age, sex, height for age z-score, weight for age z-score, plasma levels of HRP2, and preschool education of study participants.

Abbreviations: CM, cerebral malaria; CSF, cerebrospinal fluid; SMA, severe malarial anemia; CI, confidence interval.

PATHOGENS AND IMMUNITY
In Association with Case Western Reserve University and The Richard J. Fasenmyer Foundation

Supplemental Table 3. Age, sex, neurologic deficits, and cognitive outcome scores in children with CM or SMA who had CSF or plasma sST2 testing performed compared to those who did not.

Cerebral Malaria	sST2 Measured (n=249)	Not in sST2 (n=20)	P value
Age, years, median (IQR)	3.50 [2.52, 4.87]	3.41 [2.58, 4.42]	0.64
Sex, % male (n)	60.6 (151)	40.0 (8)	0.07
**Z-score for cognitive outcome at 12 months**			
**Under 5 years tests**			
Overall cognition	-0.86 [-2.17, 0.12] (119)	-1.26 [-2.01, -0.42] (12)	0.68
Attention	-0.50 [-1.29, 0.20] (124)	-0.03 [-0.58, 0.55] (12)	0.20
Associative memory	-0.46 [-0.80, -0.04] (122)	-0.42 [-0.87, -0.17] (12)	0.94
**Over 5 years tests**			
Overall cognition	-0.22 [-1.89, 1.03] (83)	3.96 [2.06, 4.22] (3)	**0.01**
Attention	0.19 [-0.84, 1.16] (83)	0.36 [0.20, 1.43] (3)	0.46
Working memory	-0.24 [-1.25, 0.75] (83)	0.63 [0.26, 1.38] (3)	0.16
**Z-score for cognitive outcome at 24 months**			
**Under 5 years tests**			
Overall cognition	-1.05 [-2.23, 0.21] (68)	-0.11 [-1.42, -0.02] (5)	0.60
Attention	-0.49 [-1.13, 0.18] (68)	-0.22 [-0.31, 0.00] (5)	0.65
Associative memory	-0.81 [-1.10, -0.25] (69)	-0.84 [-0.91, -0.63] (5)	0.75
**Over 5 years tests**			
Overall cognition	-0.71 [-1.99, 0.42] (136)	-0.29 [-0.60, 1.17] (10)	0.12
Attention	0.03 [-0.90, 0.78] (138)	0.48 [-0.14, 1.10] (10)	0.18
Working memory	-0.28 [-1.42, 0.80] (138)	-0.45 [-0.75, 0.72] (10)	0.75
**Percent of neurological deficits at:**			
Discharge	36.6% (78/213)	29.4 (5/17)	0.55
6-month follow-up	5.3% (11/209)	0% (0/14)	0.38
12-month follow-up	3.4% (7/208)	0% (0/15)	0.47
24-month follow-up	2.9% (6/205)	0% (0/15)	0.50
**Severe Malarial Anemia**	**sST2 Measured (n=193)**	**Not in sST2 (n=39)**	**P value**
Age, years, median (IQR)	2.79 [2.04, 4.35]	2.96 [2.16, 4.53]	0.91
Sex, % male (n)	60.1 (116)	61.5 (24)	0.87
**Z-score for cognitive outcome at 12 months**			
**Under 5 years tests**			
Overall cognition	-1.25 [-2.20, -0.03] (117)	-1.32 [-2.31, -0.47] (23)	0.64
Attention	-0.10 [-0.95, 0.45] (125)	-0.25 [-0.80, 0.47] (23)	0.74
Associative memory	-0.24 [-0.57, 0.33] (120)	-0.43 [-0.76, 0.17] (23)	0.13
**Over 5 years tests**			
Overall cognition	-0.24 [-0.98, 1.26] (52)	0.19 [-0.96, 0.94] (10)	0.70
Attention	0.23 [-1.04, 0.79] (54)	0.87 [-0.21, 2.24] (11)	0.29
Working memory	-0.39 [-1.53, 0.58] (54)	-0.04 [-1.37, 0.82] (10)	0.51

Fernander EM, Adogamhe P, Datta D, Bond C, Zhao Y, Bangirana P, Conroy AL, Opoka RO, John CC. Elevated plasma soluble ST2 levels are associated with neuronal injury and neurocognitive impairment in children with cerebral malaria. *Pathogens and Immunity*. 2022;7(1): 60–80. doi: 10.20411/pai.v7i1.499

PATHOGENS AND IMMUNITY
IN ASSOCIATION WITH CASE WESTERN RESERVE UNIVERSITY AND THE RICHARD J. FASENMYER FOUNDATION

**Z-score for cognitive outcome at 24 months**			
**Under 5 years tests**			
Overall cognition	-1.12 [-2.36, 0.14] (90)	-1.60 [-2.86, -1.03] (15)	0.14
Attention	-0.53 [-1.22, 0.06] (93)	-0.51 [-1.05, 0.03] (15)	0.75
Associative memory	-0.72 [-1.04, -0.12] (92)	-0.96 [-1.08, -0.22] (15)	0.19
**Over 5 years tests**			
Overall cognition	-0.20 [-1.40, 0.38] (81)	-0.31 [-1.49, 1.18] (18)	0.62
Attention	0.01 [-0.97, 0.63] (83)	0.66 [-0.95, 1.73] (18)	0.23
Working memory	-0.64 [-1.34, 0.04] (84)	-0.17 [-1.00, 0.91] (18)	0.26

For continuous variables, data presented as median [IQR] (n). Wilcoxon rank sum test used to compare age and z-scores for cognitive outcomes between children with CM or SMA who had sST2 levels measured in plasma or CSF. Chi-squared test used to compare sex and presence of neurological deficits.

Abbreviations: CM, cerebral malaria; SMA, severe malarial anemia; IQR, interquartile range.
